# Wnt signaling induces radioresistance through upregulating HMGB1 in esophageal squamous cell carcinoma

**DOI:** 10.1038/s41419-018-0466-4

**Published:** 2018-03-22

**Authors:** Yuanyuan Zhao, Jun Yi, Leilei Tao, Guichun Huang, Xiaoyuan Chu, Haizhu Song, Longbang Chen

**Affiliations:** 1 Department of Medical Oncology, Jinling Hospital, Medical School of Nanjing University, 305 Zhongshan East Road, 210002 Nanjing, Jiangsu China; 2 Department of Cardiothoracic Surgery, Jinling Hospital, Medical School of Nanjing University, 305 Zhongshan East Road, 210002 Nanjing, Jiangsu China

## Abstract

Although many articles have uncovered that Wnt signaling is involved in radioresistance, the mechanism is rarely reported. Here we generated two radioresistant cells rECA109 and rKyse150 from parental esophageal cancer cells ECA109 and Kyse150. We then found that Wnt signaling activity was higher in radioresistant cells and was further activated upon ionizing radiation (IR) exposure. In addition, radioresistant cells acquired epithelial-to-mesenchymal transition (EMT) properties and stem quality. Wnt signaling was then found to be involved in radioresistance by promoting DNA damage repair. In our present study, high-mobility group box 1 protein (HMGB1), a chromatin-associated protein, was firstly found to be transactivated by Wnt signaling and mediate Wnt-induced radioresistance. The role of HMGB1 in the regulation of DNA damage repair with the activation of DNA damage checkpoint response in response to IR was the main cause of HMGB1-induced radioresistance.

## Introduction

Esophageal cancer (EC) is the eighth most common cancer with a high mortality of the sixth most leading cause of cancer-related death worldwide^1^. According to the histopathology feathers, EC is mainly divided into esophageal adenocarcinoma (EA) and esophageal squamous cell carcinoma (ESCC). ESCC remains predominant EC especially in China. Although surgery is the main treatment of early-stage EC, radiotherapy is still the predominant treatment for the patients with late-stage EC (especially ESCC) or with no tolerance or willing of surgery^2^. Radiotherapy has many advantages in the ESCC treatment including local tumor control. However, radioresistace always happens and becomes a challenging obstacle for ESCC treatment. So it is meaningful to make out the molecular mechanisms of radioresistance and find possible strategies for increasing cellular radiosensitivity.

Active Wnt signaling is reported to induce radioresistance in several human cancers including colon cancer, nasopharyngeal cancer, glioblastoma, and head and neck cancer^3–6^. Wnt signaling always functions through the canonical pathway, that is, Wnt-induced stabilized β-catenin protein enters the nucleus and replaces T-cell factor (TCF)-associated co-repressors Groucho with coactivators like TCF/LEF, which results in the transcriptional activation of the β-catenin target genes^7,8^. So it is reasonable that it’s the β-catenin target gene that mediates Wnt-induced radioresistance.

IR kills cancer cells through several ways, among which, IR-induced DNA damage is the primary reason of cell death. Upon exposed to IR, cells generate various kinds of damaged DNA mainly including single-stranded DNA breaks (SSBs) and double-stranded DNA breaks (DSBs), the most toxic of these being DSBs^9^. DSBs are repaired by two major pathways: homologous recombination (HR) and non-homologous end joining (NHEJ). HR always happens in G2 and S phase, while NHEJ is cycle-independent. RAD51, RAD51/B/C/D, BRCA1, BRCA2, X-ray repair cross-complementing group 2 (XRCC2), and XRCC3 are responsible for HR while KU70, KU80, DNA-PKcs, DNA ligase IV, and XRCC4 are involved in NHEJ^10^.

In the context of chromatin, chromatin remodeling is necessary for DNA damage repair. The DNA-nucleosomal structure needs to be decondensed to provide DNA repair complex with access to the damage sites^11^. Moreover, phosphorylation or acetylation of histone H3 and H4 are necessary for the chromatin remodeling^12^. Many chromatin modifiers have been uncovered like chromodomain helicase DNA binding (CHD) protein, sirtuin 6 (SiRT6), ATP-dependent chromatin assembly and remodeling factor 1 (ACF1), metastasis-associated proteins 1 (MTA1), TBP-interacting protein 49 (Tip49), and Fe65^13^. HMGB1, known as chromatin-associated protein, has an essential role in DNA damage response. Upon DNA damage appearing, HMGB1 binds with DNA damage lesions, bends DNA and promotes histones H3 and H4 acetyltion, thus facilitating DNA damage recognition and other repair-related proteins entering the damage sites^14^. Furthermore, HMGB1 is also reported to be involved in DNA repair-like nucleotide excision repair (NER) and NHEJ^15^.

In this present study, we found that the Wnt signaling activity was higher in the radioresistant cell lines compared with parental esophageal squamous cell lines and inhibition of Wnt signaling could reverse the resistance to IR in the radioresistant cell lines. We next examined the molecular mechanism of Wnt-induced radioresistance and uncovered the positive correlation between Wnt signaling and HMGB1expression. β-catenin/TCF4 complex was found to transactivate HMGB1, thus promoting DNA damage repair in esophageal squamous cell carcinoma upon exposure to IR.

## Results

### Generation of radioresistant cancer cells

**Fig. 1 Radioresistant phenotype of rECA109 and rKyse150 was demonstrated in vitro and in vivo.**
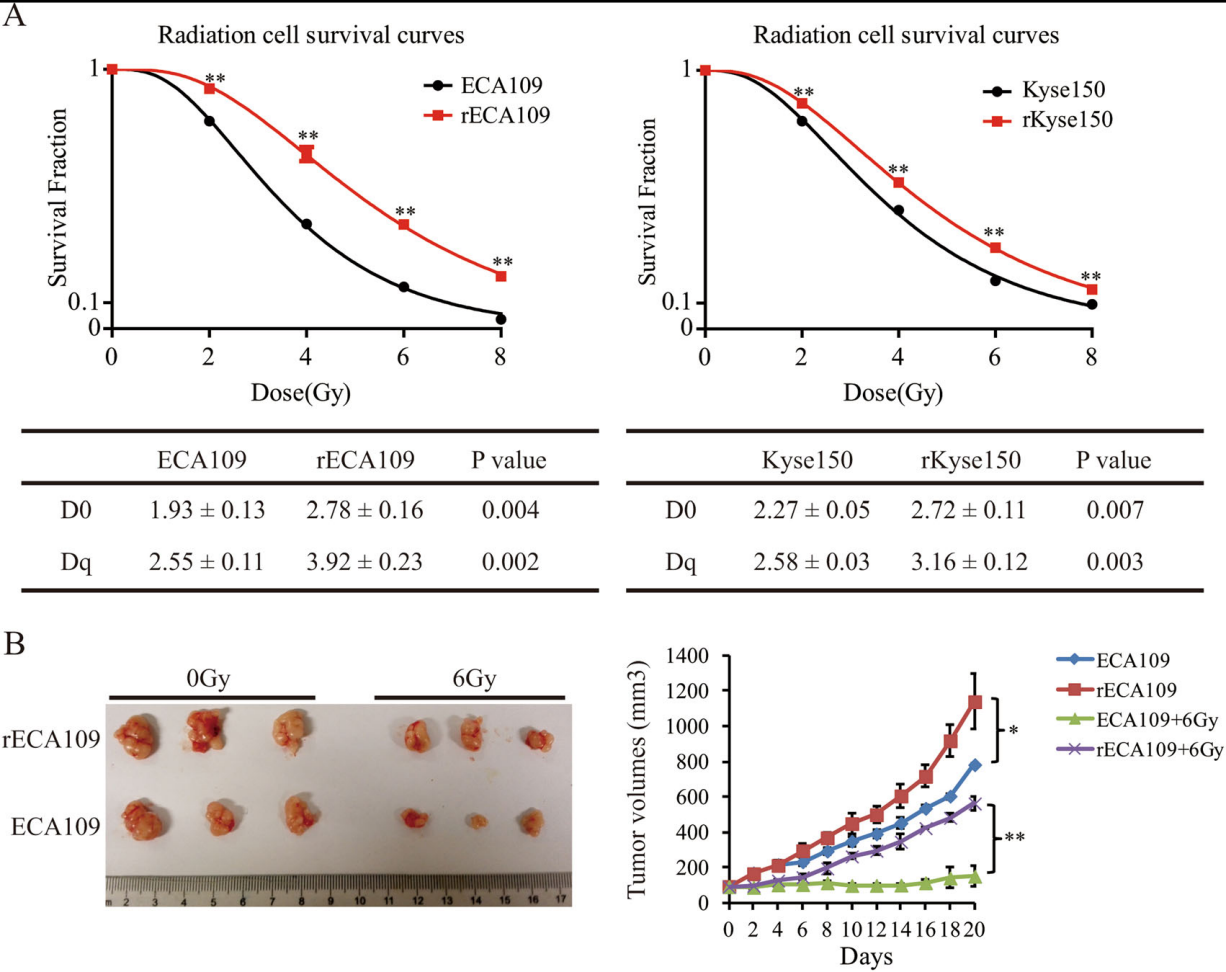
**a** Survival curves of radioresistant cells and parental cells. D0 and Dq values were calculated fitting to a multi-target model. **b** Tumor survival curves (*n* = 5). Tumor volume was recorded every other day and presented as mean ± SD. 21 days after IR, the tumors were removed. Except the mice dead after IR, three representative xenografts each group were captured and shown. *P < 0.05, **P < 0.01

To better understand the molecular mechanism of EC acquired radioresistance, we generated two radioresistant cancer cell lines from parental ECA109 and Kyse150 with radiation (2, 4, 6, 8 Gy for three times, respectively) for a total of 60 Gy. Several clones isolated from the resistant EC cell population were individually cultured and clonogenic survival assays were performed to decide the radioresistance levels of these clones. A single clone from ECA109/60 Gy and Kyse150/60 Gy population with the highest radioresistance level was picked as the radioresistant cell line called rECA109 and rKyse150, respectively^16,17^. Clonogenic survival assays indicated rECA109 and rKyse150 were more resistant to IR then parental cells (Suppl. Figure 1A). Survival curves were then analyzed using single-hit multi-target model (Fig. 1a). The D0 (Dq) values of rECA109 and rKyse150 (2.78 (3.92) and 2.72 (3.16)) were significantly higher than ECA109 and Kyse150 (1.93 (2.55) and 2.27 (2.58)), respectively (Fig. 1a).

To further demonstrate the generation of radioresistant cells, we established xenografts of ECA109, Kyse150, rECA109, and rKyse150, and exposed the tumors to IR with a single dose of 6 Gy when the volumes of tumors reached 100 mm^3^. Twenty-one days after IR, tumors were removed. From the tumor growth curves, we could see that rECA109 and rKyse150 were more resistant to IR compared with parental ECA109 and Kyse150, respectively (Fig. 1b, Suppl. Figure 1B). The tumor growth delays of rECA109 and rKyse150 (50% and 47%) were significantly lower than ECA109 and Kyse150, respectively (81% and 83%) (Supplementary Table S1). All these data demonstrated rECA109 and rKyse150 acquired radioresistant phenotype.

### Wnt signaling was activated in the radioresistant cancer cells

**Fig. 2 Radioresistant cells acquired EMT properties, characteristic associated with cancer stem cells and higher Wnt signaling activity.**
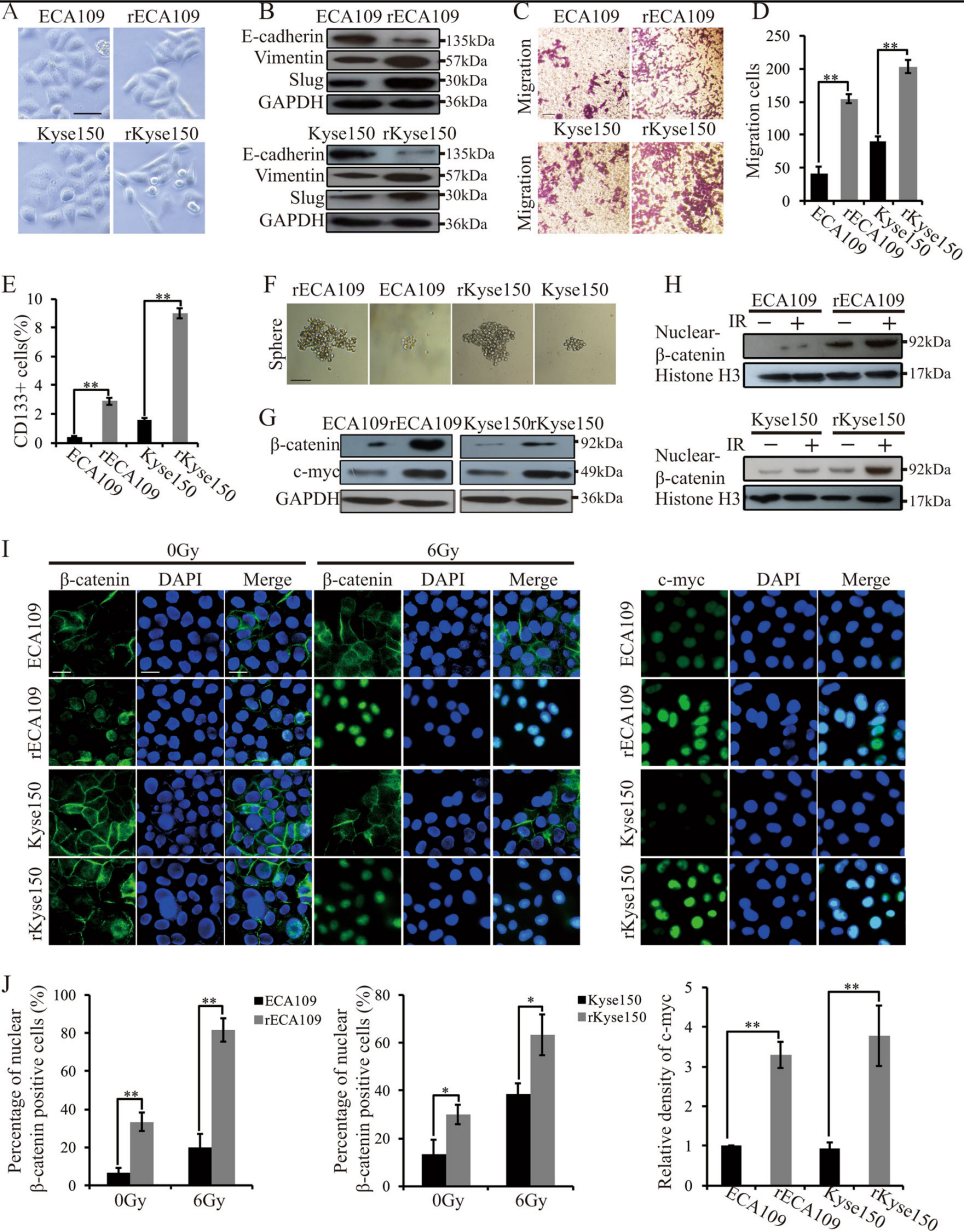
**a** Cell morphology of radioresistant cells and parental cells. The scale bars represent a distance of 50 μm. **b** Western blotting analysis of EMT representative protein. **c** Migration assay of radioresistant cells and parental cells. **d** Quantification of migration cells. Results were from **c**. Mean ± SD, *N* = 3, **P < 0.01. **e** Quantification of CD133^+^ cells. Mean ± SD, *N* = 3, **P < 0.01. **f** Representative sphere image of radioresistant cells and parental cells. Scar bars = 50 μm. **g** Western blotting analysis of total β-catenin and c-myc. **h** Western blotting analysis of nuclear β-catenin. The experiment was assessed before IR treatment or 6 h after IR (6 Gy). **i** Immunofluorescent staining of β-catenin and c-myc of ECA109, rECA109, Kyse150, and rKyse150 cells. The experiment was assessed before IR treatment and 6 h after IR (6 Gy). The scale bars represent a distance of 40 μm. **j** Quantification of nuclear β-catenin positive cells and c-myc levels through image pro plus software. Results were from **i**. Mean ± SD, *N* = 3, **P < 0.01, **P < 0.05

After the generation of radioresistant cells, we firstly noted that the morphology of rECA109 and rKyse150 was changed. The parental ECA109 and Kyse150 were round-like while rECA109 and rKyse150 were spindle shaped and smaller (Fig. 2a). Considering the correlation between EMT and the morphology transformation, we hypothesized that the radioresistant cancer cells acquired EMT properties. To confirm this hypothesis, we examined the expression levels of EMT phenotype markers by western blotting. As shown in Fig. 2b, E-cadherin was decreased while Slug and Vimentin were increased in rECA109 and rKyse150 compared with ECA109 and Kyse150. Transwell assays indicated the metastatic ability of radioresistant cells (Fig. 2c, d). In addition, rECA109 and rKyse150 acquired characteristic consistent with cancer stem cells: higher cancer stem markers CD133 expression (Fig. 2e, Suppl. Fig. 2A) and sphere formation (Fig. 2f, Suppl. Figure 2B). Considering the tight correlation between Wnt signaling and EMT and stemness, we hypothesized Wnt signaling was activated in radioresistant cells. Western blotting analysis indicated that β-catenin and Wnt target genes c-myc were upregulated in rECA109 and rKyse150 (Fig. 2g). To further confirm the activation of Wnt signaling, we analyzed β-catenin distribution. Both Western blotting and IF staining demonstrated that in the absence of IR, β-catenin is mainly distributed on the membrane of ECA109 and Kyse150. However, β-catenin partly entered the nucleus of rECA109 and rKyse150. After IR exposure, the nuclear beta-catenin was significantly increased in radioresistant cells while there were just little changes of the nuclear beta-catenin in parental cells (Fig. 2h–j). Moreover, IF staining further confirmed c-myc expression levels were higher in radioresistant cells compared with parental cells (Fig. 2i, j). All these results showed that the radioresistant cells acquired EMT and stemness properties with a higher Wnt signaling activity.

### Wnt signaling induced radioresistance by facilitating DNA damage repair

**Fig. 3 Wnt signaling promoted radioresistance via inducing DNA damage repair after IR treatment.**
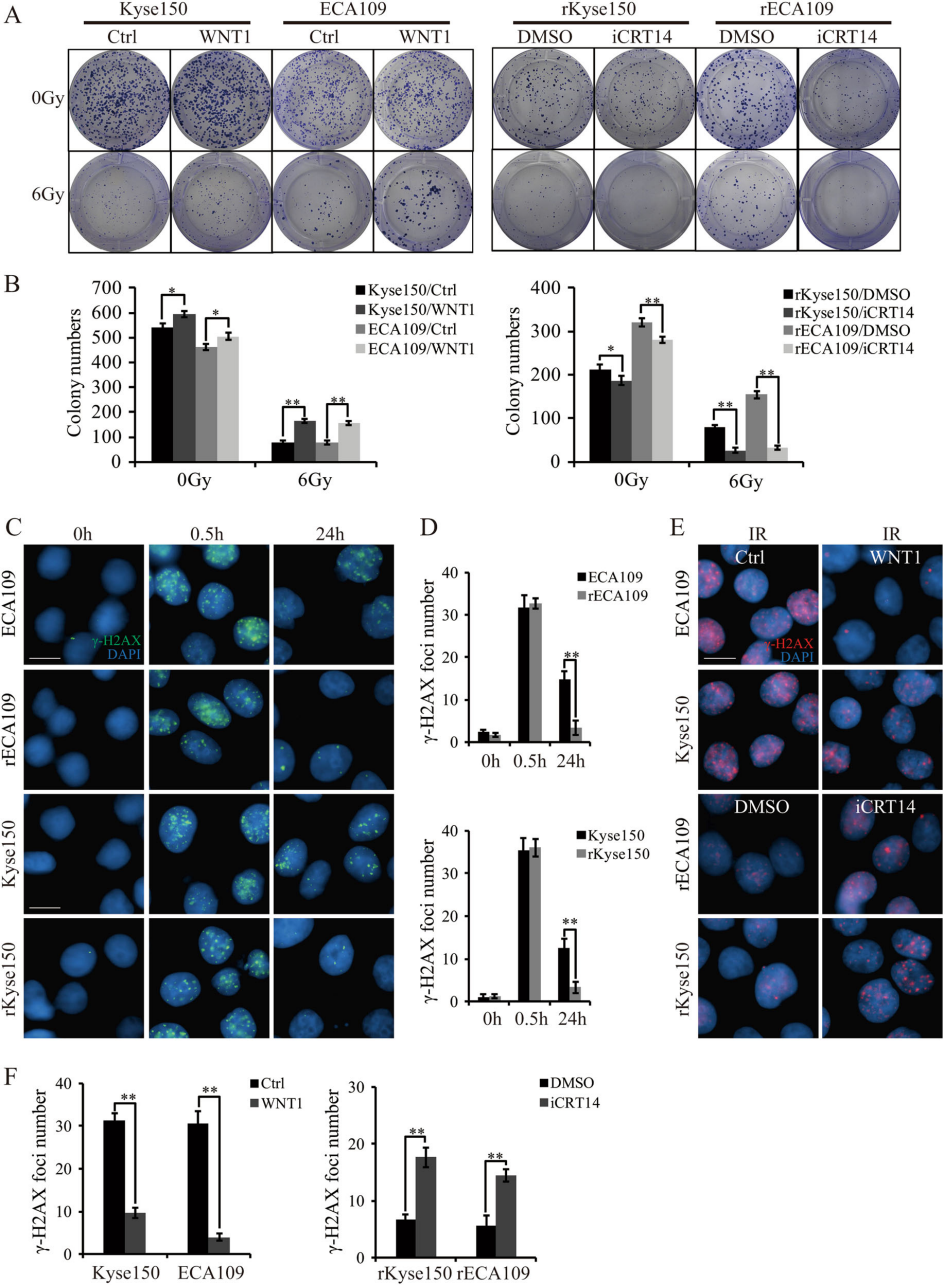
**a** Survival clonogenic assay. Parental and radioresistant cells were seeded into 6-well plates in a low density. Cells were then subjected to 0 Gy or 6 Gy of IR. The colonies were grown for 10 days in the medium containing recombinant WNT1 (100 ng/ml) (nothing as control) for ECA109 and Kyse150 and in the medium containing iCRT14 (25 μM) (DMSO as control) for rECA109 and rKyse150 cells. **b** Quantification of colony numbers from **a**. Mean ± SD, *N* = 3, *P < 0.05, **P < 0.01. **c** IF staining of phosphor-γH2AX of radioresistant cells and parental cells. Radioresistant cells and parental cells were treated with IR (6 Gy). Before IR exposure or 0.5 h, 24 h after IR exposure (6 Gy), cells were collected for IF staining for phosphor-γH2AX. Scale bars = 20 μm. **d** Mean numbers of γH2AX foci. Results were from **c**. Mean ± SD, *N* = 3, **P < 0.01. **e** IF staining of phosphor-γH2AX of cells with or without WNT1/iCRT14 pretreatment. To analyze whether DSBs repair were affected by Wnt signaling, parental cells pretreated by WNT1 (100 ng/ml) (cells without WNT1 pretreatment as control) and radioresistant cells pretreated with iCRT14 (25 μM) (cells with DMSO pretreatment as control) were subjected to IR (6 Gy). 24 h after IR (6 Gy), IF staining were performed. Scale bars = 20 μm. **f** Mean numbers of γH2AX foci. Results were from **e**. Mean ± SD, *N* = 3, **P < 0.01

As the activity of Wnt signaling in radioresistant cells was higher than in parental cells both before and after IR, we hypothesized that Wnt signaling had a role in controlling radioresistance. To confirm the hypothesis, we pretreated rECA109 and rKyse150 with iCRT14, a specific inhibitor of β-catenin-TCF interaction^3^ and subjected these cells to IR. Clonogenic assays illustrated that cells pretreated with iCRT14 followed by IR exhibited lower survival fraction compared with cells treated with IR only (Fig. 3a, b). In addition, parental cells treated with WNT1^18^ and receiving IR thereafter, displayed higher survival rates than cells exposed to IR only (Fig. 3a, b). The result uncovered the important role of Wnt signaling in preventing cells dying of IR.

Although irradiation kills cells by several mechanisms, DNA damage is still the primary reason of cell death. To compare DNA damage levels between parental and radioresistant cells after IR, we subjected them to IR and analyzed phospho-γH2AX levels, a marker of DNA DSBs, by IF staining^3^. It was shown that at the early time point (0.5 h after IR), there was no difference in the DNA damage foci formation. However, at the later time point (24 h after IR), parental cells still exhibited sustained γH2AX foci while radioresistant cells basically recovered from DNA damage (Fig. 3c, d). In addition, we tested another DSB biomarker, mediator of DNA damage checkpoint protein 1 (MDC1) whose change was consistent with γH2AX foci (Suppl. Fig. 3A, Suppl. Figure 3B). Given the tight association between Wnt signaling and radioresistance, it is reasonable of Wnt signaling involving DNA damage repair. We then treated parental cells and radioresistant cells with WNT1 and iCRT14, respectively, and exposed them to IR subsequently. IF staining showed that parental cells pretreated with WNT1 showed decreased γH2AX foci and MDC1 expression, whereas radioresistant cells pretreated with iCRT14 displayed increased γH2AX foci and MDC1 expression (Fig. 3e, f, Suppl. Fig. 3C, Suppl. Figure 3D).

### Wnt signaling promoted chromatin remodeling and the activation of DNA damage checkpoint response

**Fig. 4 Wnt signaling promoted chromatin remodeling and the activation of DNA damage checkpoint response after IR treatment.**
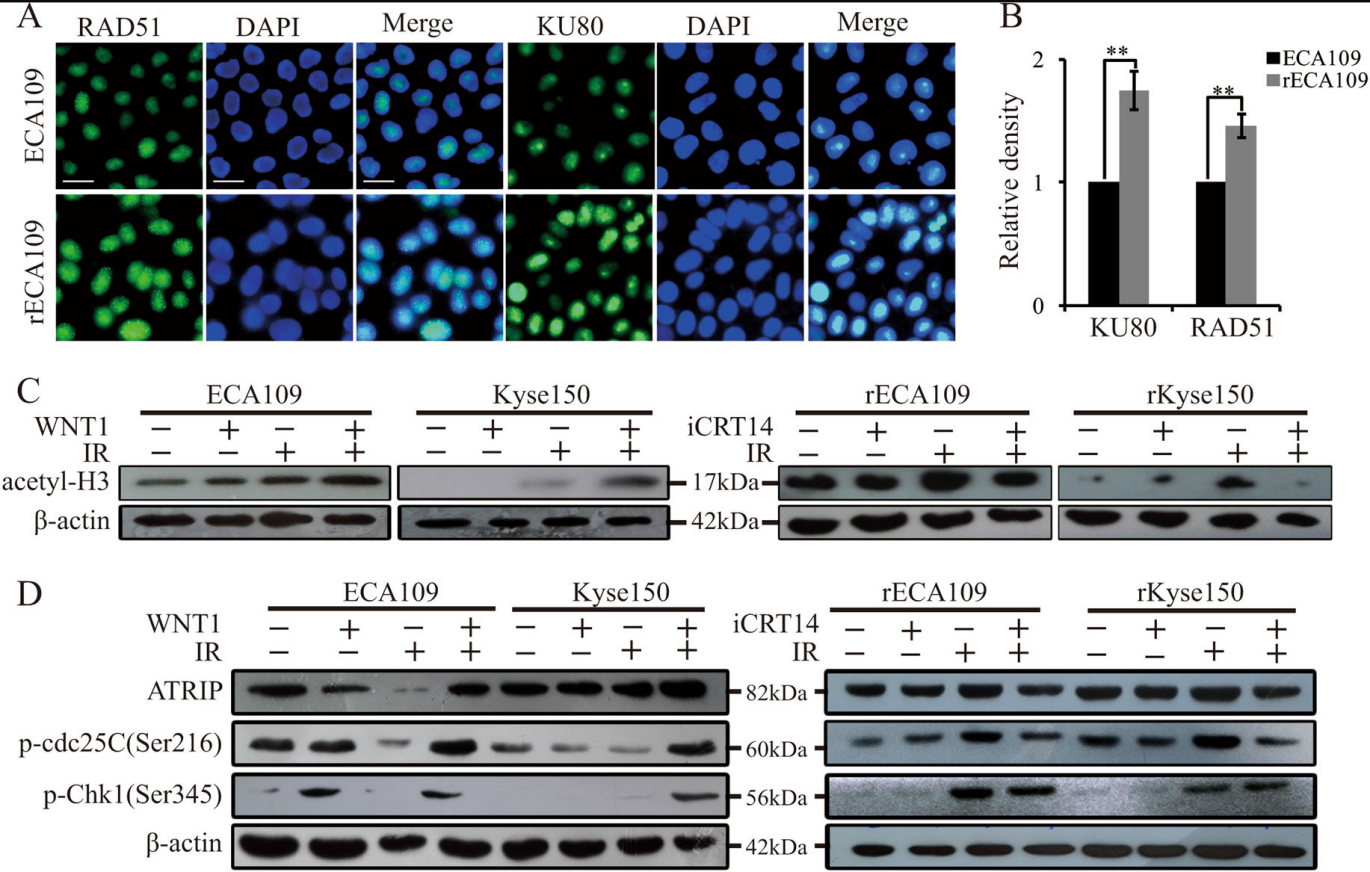
**a** IF staining of KU80 and RAD51. ECA109 and rECA109 cells were collected to perform IF staining 6 h after IR (6 Gy). Scale bars = 40 μm. **b** Quantification of KU80 and RAD51 by image pro plus software. Results were from **a**. Mean ± SD, *N* = 3, **P < 0.01. **c** Western blotting analysis of acetyl-H3 (Lys9). Parental cells with or without WNT1 and radioresistant cells with or without iCRT14 pretreatment were subjected to IR (6 Gy). Before IR or 4 h after IR, Western blot was performed. **d** Western blotting analysis of representative proteins of DNA damage checkpoint response. Parental cells with or without WNT1 pretreatment and radioresistant cells with or without iCRT14 pretreatment were subjected to IR (6 Gy). Before or 0.5 h after IR (6 Gy), total proteins were extracted and then immunoblotted

Next we sought to understand the mechanism of Wnt-induced DNA damage repair. Our experiments showed that after IR treatment, HR-related protein RAD51 and NHEJ-related protein KU80 were slightly upregulated in rECA109 and rKyse150 cells (Fig. 4a, b). With iCRT14 pretreatment, we found that in the absence of IR, iCRT14 inhibited NHEJ-related factors expression and promoted HR-related proteins expression (Suppl. Fig. 4A). However, in response to IR, iCRT14 inhibited both HR and NHEJ-related proteins expression (Suppl. Figure 4B), which indicated that Wnt signaling not only influenced the process of NHEJ directly, but also controlled the other process of DNA damage repair. So we hypothesized that Wnt signaling may have a role in the initial of DNA damage repair mainly including DNA damage recognition and chromatin access to the DNA damage sites. Deng et al.^9^ has uncovered the role of β-catenin in upregulating Mre11 (a component of DNA damage recognition complex) following IR. Here we noted that Wnt signaling also affected chromatin remodeling after DNA damage. As the acetylation of histones H3 is a representative biomarker of chromatin access to the DNA damage sites, we tested the acetyl-H3 levels in cells treated by iCRT14 or WNT1 both before and after IR. We observed that both WNT1and iCRT14 were not required for basal-level expression of acetyl-H3. However, WNT1 increased acetyl-H3 levels while Wnt inhibitor decreased it after IR (Fig. 4c). The result reminded us of the role of Wnt signaling in promoting chromatin remodeling and then inducing radioresistance.

In addition, DNA damage repair is always accompanied with the activation of DNA damage checkpoint response as temporal cell cycle arrest is necessary to provide a window for DNA repair^19^. Cell cycle analysis indicated that there was no significant difference in cell cycle distribution between unirradiated radioresistant cells and parental cells. However, the fraction of cells in G2/M phase significantly increased in radioresistant cells while there was a slight increase in parental cells 0.5 h after IR, which indicated the weak ability of parental cells halting cell cycle progression after IR (Suppl. Fig. 4C). Wnt-regulated c-myc was reported to be essential for the activation of ATM and Chk1/2^20,21^. To further determine whether there existed difference of the activation of DNA damage checkpoint responses between radioresistant cancer cells and parental cells, we examined the expression levels of activating phosphorylated Chk1, Chk2, cdc25C, and ATRIP by western blotting after IR treatment. As shown in Suppl. Figure 4D, rECA109 and rKyse150 cells showed preferential activation of DNA damage checkpoint responses upon exposure to IR relative to parental cells. And WNT1 promoted preferential activation of DNA damage response while iCRT14 hindered it (Fig. 4d). All these experiments demonstrated that the activation of Wnt signaling resulted in preferential chromatin access and the activation of DNA damage checkpoint response upon exposure to IR, thus inducing radioresistance.

### HMGB1 was transactivated by Wnt signaling

**Fig. 5 HMGB1 was transactivated by β-catenin/TCF4 complex.**
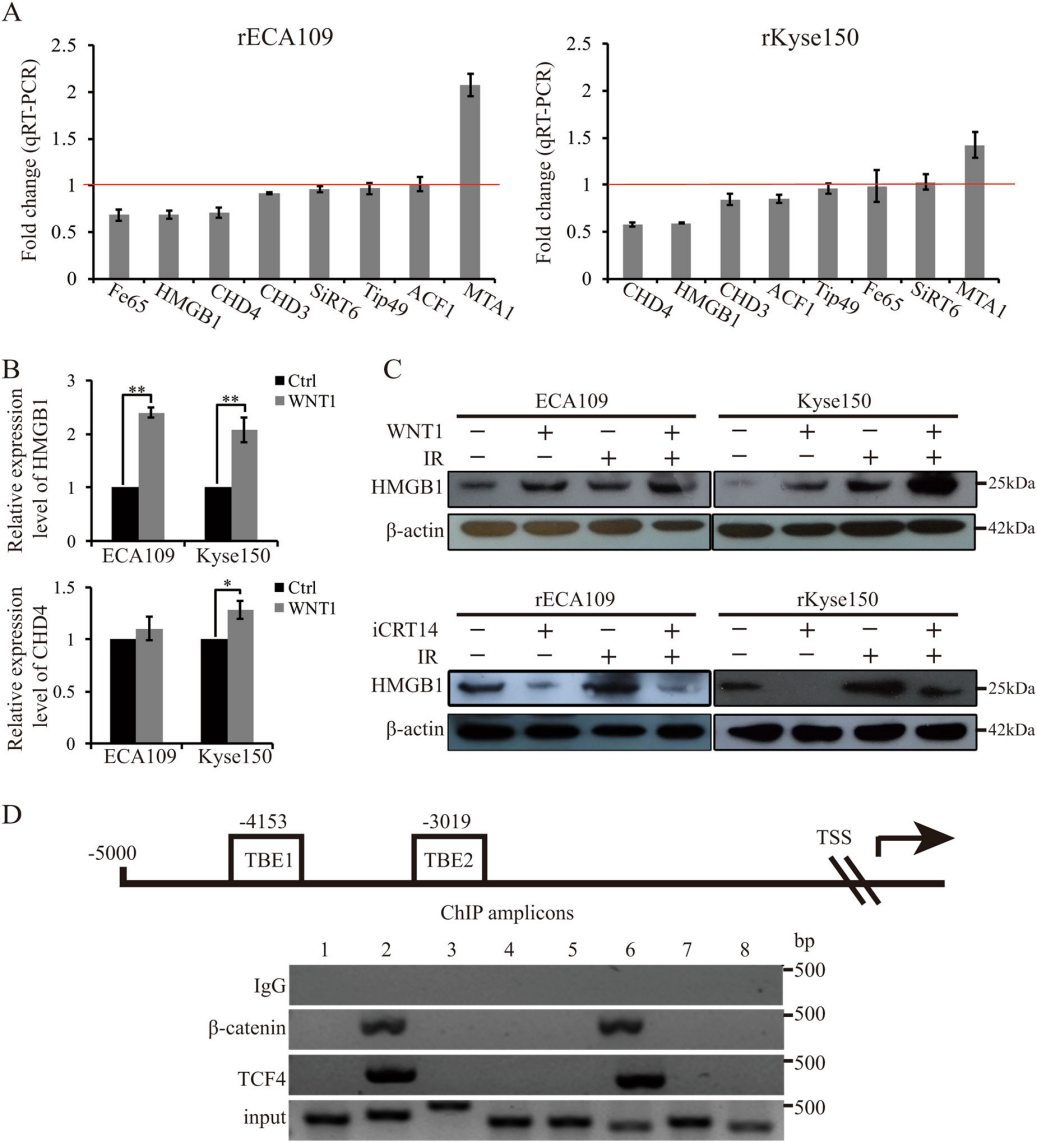
**a** qRT-PCR analysis of chromatin modifiers’ fold changes after the inhibition of Wnt signaling. rECA109 and rKyse150 cells were treated with iCRT14 (25 μM for 24 h) and analyzed for expression of chromatin modifiers using qRT-PCR. **b** qRT-PCR analysis of HMGB1 and CHD4. 24 h after treatment of WNT1 protein, qRT-PCR was performed to analyze the expression of HMGB1 and CHD4. Mean ± SD, *N* = 3, *P < 0.05, **P < 0.01. **c** Western blotting analysis of HMGB1. Western blottings were performed to analyze the expression levels of HMGB1 after WNT1 or iCRT14 treatment with or without IR. **d** ChIP assay of rKyse150 cells. β-catenin/TCF4 transcriptional complex occupies HMGB1 promoter. ChIP amplicons (1–8) were detected by ChIP-PCR

Next, we explored the molecular mechanism of Wnt-mediated chromatin remodeling after IR. As canonical Wnt signaling mainly works by transactivating target genes, we performed qRT-PCR to test the change levels of several histone modifiers after iCRT14 treatment. The results showed that the transcriptional levels of HMGB1 and CHD4 were downregulated both in rECA109 and rKyse160 after iCRT14 treatment (Fig. 5a). In addition, we further confirmed that WNT1 treatment promoted HMGB1 expression while there was no significant difference of CHD4 levels between cells treated with WNT1and control group (Fig. 5b). So HMGB1 was chosen to be a possible target of Wnt signaling. The result of western blotting analysis of HMGB1 was consistent with the result of qRT-PCR (Fig. 5c). By the analysis of promoter sequence of HMGB1, we found two putative TCF binding elements (TBEs, 5′(A/T)(A/T)CAAAG3′)^22^, which located at −4153 (TBE1) and −3019 (TBE2) (Fig. 5d). ChIP assay indicated that the HMGB1 genomic DNA promoter fragment (−4146 to −3801; −3099 to −2793) could be amplified by in groups using TCF4 or β-catenin antibody to immunoprecipitate chromatin, which indicated that β-catenin/TCF4 heterodimer could directly bind to the promoter of HMGB1 (Fig. 5d).

### HMGB1 had a role in radioresistance

**Fig. 6 HMGB1 promoted cancer radioresistance.**
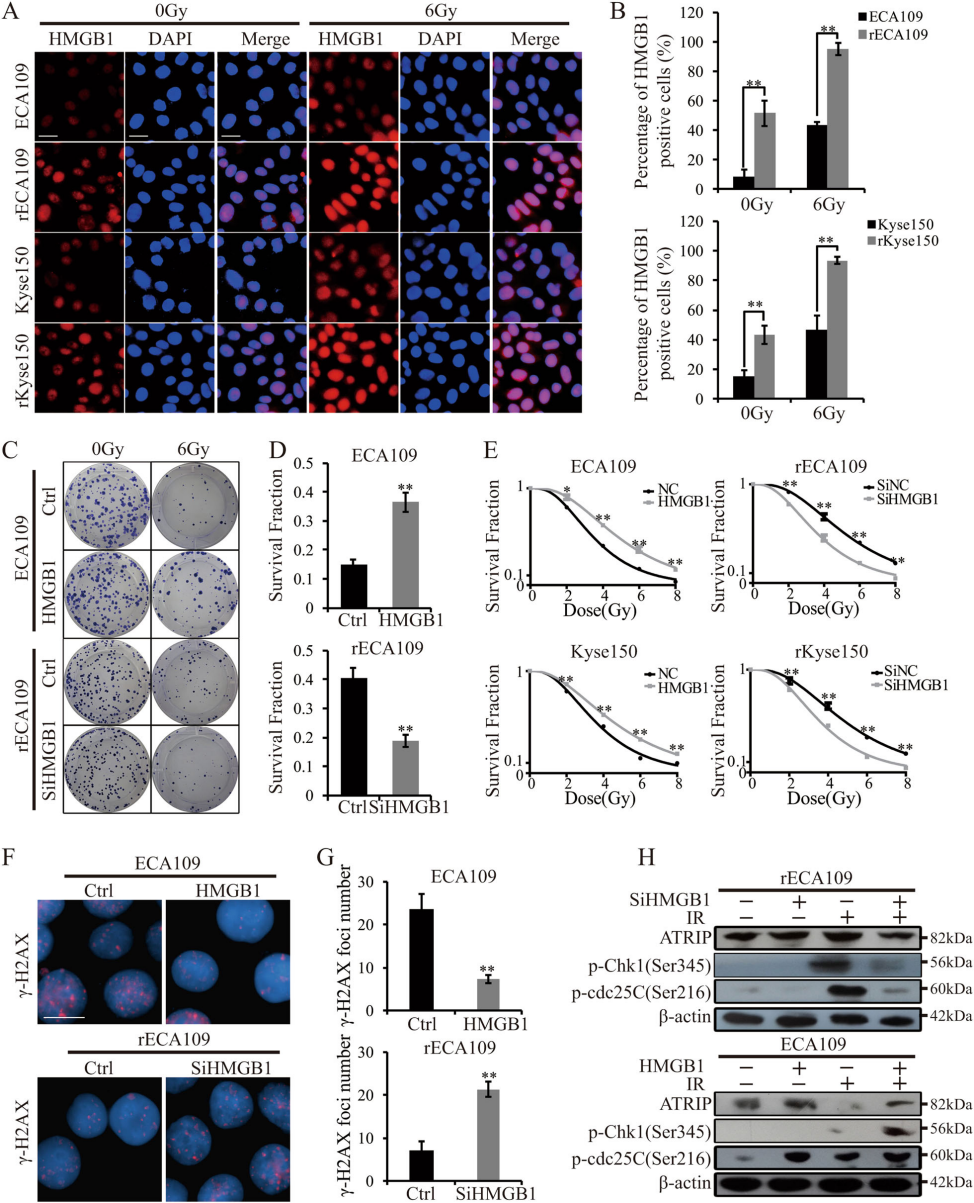
**a** IF staining of HMGB1. The experiment was assessed before IR treatment and 6 h after IR (6 Gy). Scale bars = 40μm. **b** Quantification of HMGB1 positive cells. Results were from **a**. Mean ± SD, *N* = 3, **P < 0.01. **c** Survival clonogenic assay. ECA109/HMGB1 (with ECA109/NC as control) and rECA109/SiHMGB1 (with rECA109/SiNC as control) cells were untreated or treated with 6 Gy of IR. Colonies were grown for 10 days. **d** Survival fractions of ECA109/HMGB1, ECA109/NC, rECA109/SiHMGB1, and rECA109/SiNC cells after IR treatment. Results were from **c**. Survival fraction = number of colonies formed/number of cells seeded × plating efficiency of the control group where plating efficiency was calculated as ratio between colonies observed and number of cells plated in the absence of IR. Mean ± SD, *N* = 3, **P < 0.01. **e** Survival curves. Cells were exposed to 0, 2, 4, 6, 8 Gy after stable plenti/HMGB1 transfection or plenti/SiHMGB1 transfection. Mean ± SD, *N* = 3, *P < 0.05, **P < 0.01. **f** IF staining of phosphor-γH2AX. Stable-transfected ECA109 cells (plenti/HMGB1 and plenti/NC) and rECA109 cells (plenti/SiHMGB1 and plenti/SiNC) were collected to analyze the DSB levels 24 h after IR using IF staining. Scale bars = 20μm. **g** Mean numbers of γH2AX foci. Results were from **f**. Mean ± SD, *N* = 3, **P < 0.01. **h** Western blotting analysis of representative proteins of DNA damage checkpoint response. ATRIP, p-cdc25C, and p-Chk1 were assessed when HMGB1 was upregulated or downregulated using Western blotting

The role of HMGB1 in chromatin remodeling and NHEJ determinated it involving in radioresistance. It was reported that HMGB1 regulated radiosensitivity of breast cancer and bladder cancer^15,23^. In our experiments, IF staining revealed that in the absence of IR, HMGB1 expression was higher in rEC109 and rKyse15. In response to IR, HMGB1 expression was increased both in parental and radioresistant cells. In addition, the expression levels of HMGB1 in radioresistant cells were significantly higher than parental cells after IR (Fig. 6a, b). The western blotting result was consistent with the result of IF staining (Suppl. Fig. 5A). To determine the role of HMGB1 in radioresistance, we stably transfected parental cells and radioresistant cells with HMGB1 expression vector or HMGB1-specific siRNA, respectively, with the negative control of the empty vector or control siRNA (NC, SiNC). HMGB1 expression was changed after vector transfection shown in Suppl. Figure 5B. Clonogenic survival assays showed that HMGB1 overexpression made parental cells resistant to irradiation and HMGB1 inhibition radiosensitized rECA109 and rKyse150 (Fig. 6c–e). D0 and Dq values were significantly increased when HMGB1 was upregulated while significantly decreased when HMGB1 was downregulated (Supplementary Table S2). Next, we sought to understand the molecular mechanism of HMGB1-induced radioresistance. As HMGB1 is a chromatin modifier, we hypothesized that HMGB1 had a role in DNA damage repair. Upregulation of HMGB1 promoted cancer recovering from IR-induced DNA damage while downregulation of HMGB1attenuated DNA damage repair (Fig. 6f, g, Suppl. Fig. 5C, Suppl. Figure 5D). In addition, considering the correlation between histone modifiers and DNA damage checkpoint response^13^, we assessed several DNA damage checkpoint proteins and found HMGB1 induced preferential activation of DNA damage checkpoint response (Fig. 6h). Above all, HMGB1 promoted radioresistance through facilitating DNA damage repair with the preferential activation of DNA damage checkpoint response.

### Wnt signaling-induced radioresistance partly depended on HMGB1

**Fig. 7 HMGB1 partly mediated Wnt-induced radioresistance.**
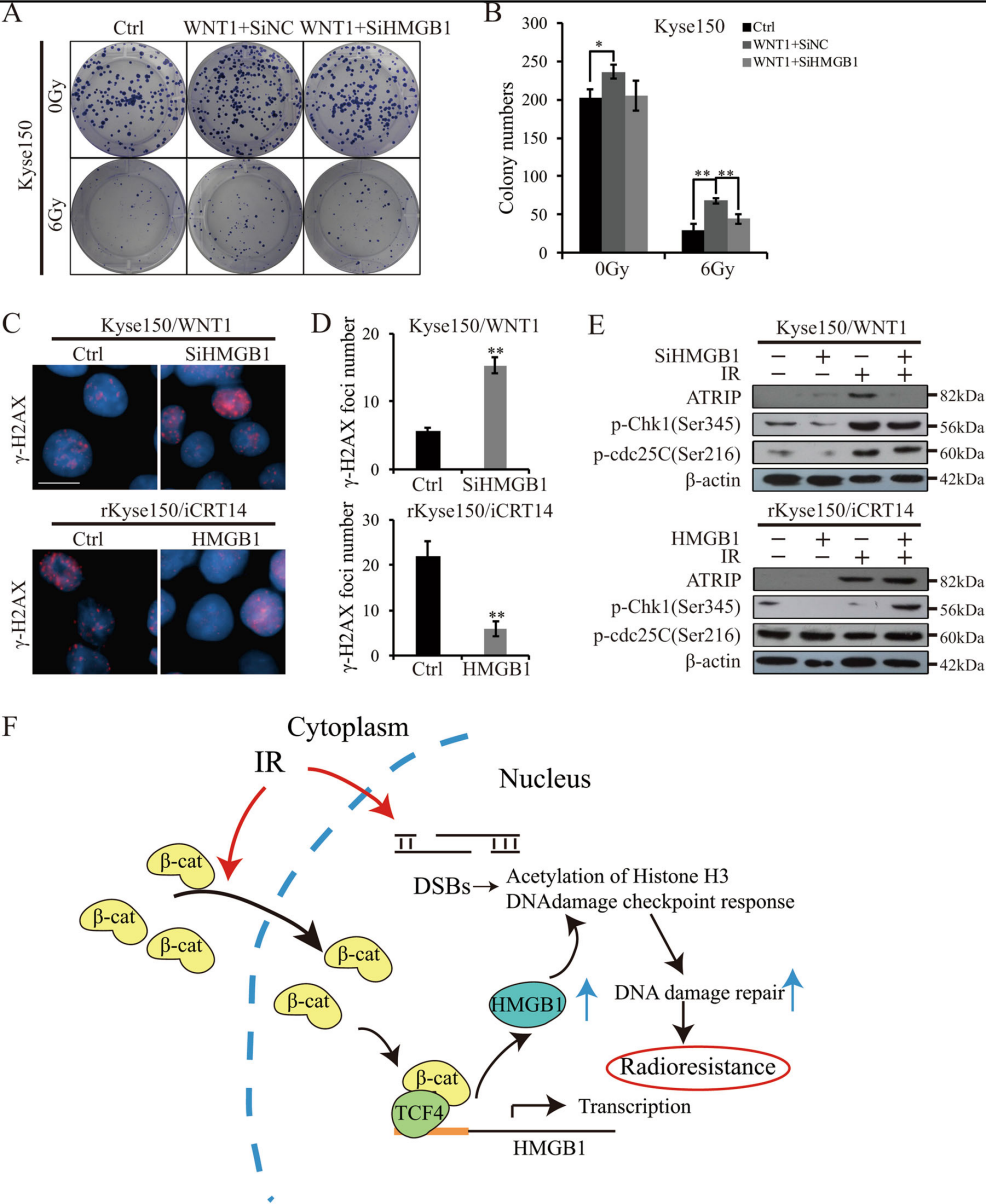
**a** Survival clonogenic assay. Kyse150 cells stably transfected with plenti/SiHMGB1 (NC as control) treated with WNT1 were collected to perform clonogenic assays with or without IR. **b** Quantification of colony numbers from **a**. Mean ± SD, *N* = 3, *P < 0.05, **P < 0.01. **c** IF staining of phosphor-γH2AX. 24 h after WNT1 treatment for Kyse150/SiHMGB1 and Kyse150/SiNC cells and 24 h after iCRT14 treatment for rKyse150/HMGB1 and rKyse150/NC cells, cells were exposed to IR. 24 h after IR, IF staining of phosphor-γH2AX were performed to assess the DNA damage levels. Scale bars = 20 μm. **d** Mean numbers of γH2AX foci. Mean ± SD, *N* = 3, *P < 0.05, **P < 0.01. The results were from **c**. e Western blotting analysis of representative proteins of DNA damage checkpoint response. The expression levels of ATRIP, p-cdc25C, and p-Chk1in Kyse150/SiHMGB1 (Kyse150/SiNC) cells with WNT1 treatment and rKyse150/HMGB1 (rKyse150/NC) cells with iCRT14 treatment were assessed via western blotting. **f** Schematic diagram. Upon IR exposure, β-catenin enters the nucleus and binds with TCF4, thus transactivating HMGB1. HMGB1 then acting as the chromatin modifier, promotes the acetylation of histones H3 and the activation of DNA damage response, which induces cellular radioresistance

As HMGB1 was transactivated by beta-catenin/TCF complex, we hypothesized that it was HMGB1 that mediated Wnt signaling-induced radioresistance. Stably Kyse150/siHMGB1 and rKyse150/HMGB1 cells were generated (Suppl. Figure 6a). Clonogenic assay illustrated that the inhibition of HMGB1 partly resensitized EC cells pretreated by WNT1 (Fig. 7a, b). Furthermore, cells pretreated by WNT1 still exhibited DSBs in response to IR when HMGB1 was inhibited. Also, iCRT14-induced the attenuation of DNA damage repair after IR was partly reversed by upregulating HMGB1 (Fig. 7c, d, Suppl. Figure 6B, Suppl. Fig. 6C). WB analysis indicated downregulation of HMGB1 inhibited the activation of DNA damage checkpoint response in WNT1-pretreated Kyse150 cells after IR treatment (downregulation of ATRIP and p-Chk1). In addition, upregulation of HMGB1 reversed iCRT14-induced the attenuated activation of DNA damage checkpoint response in rKyse150 cells after IR treatment (upregulation of ATRIP, p-Chk1 and p-cdc25C) (Fig. 7e). However, p-cdc25C levels were not changed when HMGB1 was inhibited in WNT1-pretreated Kyse150 cells. This indicated us that there possibly existed other downstream targets of Wnt signaling as to promote the activation of DNA damage checkpoint response. We would explore the question in the future. All these results indicated that Wnt signaling-induced radioresistance partly depended on HMGB1.

In conclusion, upon IR exposure, β-catenin entered the nucleus and transactivated HMGB1 expression. HMGB1 then acted as chromatin modifier for increasing chromatin accessibility and activated DNA damage checkpoint response (Fig. 7f). In this way, DNA damage repair machine was initiated and protected cancers from the damage of IR.

## Discussion

Although radiotherapy is a common treatment of ESCC, radioresistance always happens and limits the application of radiotherapy. So it is necessary to make out the mechanism of radioresistance. Although the mechanisms of cancer radioresistance are complicated and poorly understood, strategies for promoting radiosensitization have achieved success. In phase 3 clinical trials, radiation in combination with the EGFR inhibitor cetuximab has been proved to achieve a survival benefit of 10% in head and neck squamous cell carcinoma (HNSCC)^24^. In preclinical models, PI3K/mTOR inhibitor has turned out HNSCC radiosensitization^25^. Here we generated two radioresistant cell lines and uncovered that the Wnt signaling was activated in radioresistant cells. The next experiments demonstrated that Wnt signaling was tightly associated with cellular response to IR and the inhibitor targeting Wnt signaling increased radiosensitivity.

The molecular mechanism of Wnt-induced radioresistance mainly focuses on the relationship between Wnt signaling and cancer stem cells^26,27^. And we turned out that radioresistant cells with high Wnt signaling activity were endowed with stemness. However, cancer stem cells induce radioresistance dependent on its role in promoting DNA damage repair, reactive oxygen species (ROS) scavenging and inhibiting apoptosis^28^. So it’s more intuitive to examine the relationship between Wnt signaling and DNA damage repair, ROS scavenging and apoptosis. As DNA damage is the primary reason of IR-induced cell death, we firstly focused on the relationship between Wnt signaling and DNA damage repair. Wnt signaling was demonstrated to be involved in p53-dependent and p53 independent DNA damage repair pathway. All these research mainly focused on the effect of Wnt signaling on the repair process of repair (mainly NHEJ)^3,6,29^. In our results, we also demonstrated that Wnt signaling had an essential role in chromatin remodeling and DNA damage checkpoint response. Chromatin remodeling is necessary for the access to the damage sites and DNA damage checkpoint response is required to provide a window for DNA damage repair. So it is meaningful to target them for promoting radiosensitization. Apart from DNA damage repair, oxidative stress regulation and apoptosis have an important role in deciding the consequence of IR. It was demonstrated that inhibition of β-catenin caused a decreased expression of the hydrogen peroxide (H2O2) detoxifying enzyme catalase and led to the accumulation of reactive oxygen species (ROS) upon exposure to IR^30^. Furthermore, β-catenin can function as an important molecular defeating against oxidative stress through controlling the balance between TCF (mainly proliferative) and Forkhead box (FOXO) (mainly stress response) signaling pathway. FOXO family is an important ROS scavenger via induction of anti-oxidant proteins like MnSOD, catalase and GADD45. Cells could be protected from ROS induced by radiation through the upregulation of FOXO^13^. In addition, Wnt-1-induced secreted protein (WISP-1), as an important downstream target gene of Wnt1/β-catenin signaling, was reported to prevent cancer from P53-mediated apoptosis and inhibit the mitochondrial release of cytochrome c and elevate the expression of anti-apoptotic protein Bcl-XL^31^. Moreover, it was reported that Wnt signaling promotes survivin expression in colon cancer^32^. In our study, we found that Wnt signaling had a role in inhibiting ROS generation and apoptosis (data were not shown), which may be a focus of the next research.

Histone modifiers were reported to be involved in radioresistance. For example, histone acetylation by CBP/p300 promoted radioresistance by facilitating the recruitment of the NHEJ factors to the damage sites^33^. ACF1 complex increased overall efficiency of repair including NHEJ and HR after IR^34^. To determine the mechanism of Wnt-induced Histones H3 acetylation, we assessed several histone modifiers expression changes after the inhibition of Wnt signaling. We firstly found that HMGB1 was regulated by Wnt pathway. Upon inhibition of Wnt signaling, HMGB1 was downregulated and WNT1 upregulated HMGB1. ChIP assays indicated that HMGB1 was transactivated by β-catenin/TCF4 complex. In the next experiments, HMGB1 was significantly upregulated in radioresistant cells. Furthermore, blocking HMGB1 sensitized radioresistant cells to IR with the inhibition of DNA damage repair while overexpressing HMGB1 promoted parental cells radioresisance with the enhancement of DNA damage repair. Moreover, blocking HMGB1 partly attenuated WNT1-induced radioresistance and DNA damage repair. These results strongly indicated that Wnt-induced radioresistance was partly dependent on HMGB1.

The distribution of HMGB1 both in the cytoplasm and nucleus of cancer cells and in the tumor microenvironment decides its role in regulating other cellular physiology process apart from DNA damage repair^35^. HMGB1 cytoplasm translocation and extracellular release are induced in response to IR^36^. Released HMGB1 has been reported to bind with the receptor for advanced glycation end products (RAGE), promoting autophagy and inhibiting apoptosis in stressed cancer cells^37^. HMGB1 has an essential role in balancing autophagy and apoptosis. However, Jinghua Wu put forward a contrary opinion that HMGB1 increased apoptosis rates through affecting p53 expression in response to IR in hepatocellular carcinoma^38^. The role of HMGB1 needs further exploration.

In this article, we uncovered the correlation between Wnt signaling and DNA damage repair and demonstrated HMGB1-mediated Wnt-induced radioresistance. So Wnt inhibitors hold a great potential for acting as a combination treatment with radiotherapy.

## Materials and methods

### Mammalian cell culture and materials

ECA109 and Kyse150 were purchased from the Tumor Cell Bank of the Chinese Academy of Medical Science (Shanghai, China) and cultured in RPMI 1640 medium and DMEM, respectively, containing 10% fetal bovine serum and ampicillin and streptomycin at 37 °C in a humidified atmosphere of 95% air and 5% CO_2_.

### Establishment of radioresistant cells

The parental cells ECA109 and rECA109 received 2 Gy irradiation when the cells achieved 50–60% confluence. After IR treatment, the cells were cultured, split1:3 and receive IR again (2 Gy × 3, 4 Gy × 3, 6 Gy × 3 and 8 Gy × 3 times).When the total radiation doses reached 60 Gy, several clones isolated from the resistant EC cell population were individually cultured. Three months after the termination of IR, clonogenic cell survival assays were preformed to decide the radiresistance levels of these clones. The clones named rECA109 and rKyse150 separately thereafter were then picked to be the radioresistant cell lines.

### Clonogenic cell survival assays

Clonogenic cell survival assays were performed based on the routinely performed protocol. Briefly, before IR exposure, cells were subjected to different stimulation including recombinant WNT1 treatment (abcam; UK), iCRT14 treatment (MCE; USA), plenti-HMGB1 or SiHMGB1 transfection. Then the cells were seeded into six-well plates (800 cells/well) and received IR of different doses (0, 2, 4, 6, 8 Gy) and grown for 10 days. Finally, cells were fixed and stained with crystal violet. Colonies containing more than 50 cells were identified as surviving colonies. Surviving fraction (SF) was estimated by the following formula: SF = number of colonies formed/number of cells seeded × plating efficiency of the control group where plating efficiency was calculated as ratio between colonies observed and number of cells plated. Dose–response clonogenic survival curves were plotted on a log-linear scale. At least three cell concentrations were used for each radiation dose. The average data were fitted into single-hit multi-target formula: *S* = 1 − (1 − *e*^−D/D0^)^*N*^, where *S* is the fraction of cells surviving a dose, D0 called the “mean lethal dose”, is the dose on the straight-line portion of the survival curve to decrease the survival to 37%. The “quasi-threshold dose” or Dq, which is the intercept of the extrapolated high dose, was also calculated. *N* is referred to the extrapolation number, which is a parameter to measure the width of shoulder of the survival curve.

### Tumor xenograft experiments

Female BALB/c nude mice (CByJ.Cg-Foxn1nu/J) were purchased from Model Animal Research Center of Nanjing University and maintained under controlled temperature and humidity, and a 12-hours light-dark cycle, with sterile food and water ad libitum. All mice were 4–6 weeks old. All the animal experiments were performed according to the institutional guidelines and approved by the Ethical Review Committee of Comparative Medicine, Jinling Hospital, Nanjing, China. Nude mice were subcutaneously injected with 5 × 10^6^ cells. A total of 40 mice were divided into 8 groups (5 mice/per group), that is: ECA109, rEA109, Kys150, and rKyse150 xenografts transplantation with or without IR treatment. When the average tumor volume reached approximately 100 mm^3^ (about 10 days after tumor transplantation), groups of receiving IR treatment were irradiated with a single 6 Gy dose of IR. Twenty-one days after IR, all the tumors were removed. Subcutaneous tumor volumes were measured every other day by caliper and tumor volumes were calculated by the formula: tumor volume = 0.5 × length × width × width. Tumor growth delay was calculated using the formula: tumor growth delay = (tumor volumes without IR − tumor volumes with IR)/tumor volumes without IR.

### Cell migration assay

Overall, 2 × 10^4^ cells in serum-free media were placed into the upper transwell chamber. Media containing 10% FBS was added into the lower chamber. Following 48 h incubation, cells remaining in upper membrane were wiped off, whereas cells that migrated were fixed in methanol, stained with 0.1% crystal violet and counted under a microscope. Three independent experiments were carried out.

### Tumor sphere formation assay

Cells were seeded in triplicate onto 6-well ultra-low attachment plates (500 cells/well) in serum-free DMEM/F-12 supplemented with 20 ng/ml epidermal growth factor, 20 ng/ml basic fibroblast growth factor and 2% B27 (Invitrogen; USA). After 10 days of culture, the number of tumor spheres formed (diameter > 100 µm) was counted under the microscope.

### Cell cycle analysis

Cell cycle distribution was analyzed before, and 0.5 h after irradiation at 6 Gy. Cell membranes were permeabilized at -20°C overnight by 70% ethanol. Subsequently, cells were then treated with RNase A (Keygen, China) for 30 min at 37 °C and stained with propidium iodide (Keygen, China) for 20 min at room temperature. DNA content was measured by flow cytometry and analyzed using the FlowJo software package.

### qRT-PCR

For RNA extraction, cells were processed using Trizol reagent (Invitrogen; USA). RNA was subjected to reverse transcription using SuperScript II (Invitrogen; USA). Next, complementary DNA was used for gene expression analysis using qRT-PCR. Primers of target genes were shown in Supplementary Table S3.

### Western blotting

For total protein extraction, cells were lysed by a lysis buffer containing protease inhibitor cocktail (Roche; USA) and PMSF (Roche; USA). To separate nuclear fraction, we used NE-PER nuclear and cytoplasmic extraction kit (Piece, Rockford, USA) according to the manufacturer’s instruction^39^. Western blotting was then performed according to the routinely protocol including protein separation, membrane-transferring, block and primary and secondary antibody incubation. The primary antibodies were against β-actin (abcam; 1:5000; UK), GAPDH (abcam; 1:5000; UK), c-myc (proteintech; 1:1000; USA), RAD51 (abcam; 1:10000; UK), KU80 (abcam: 1:10000; UK), β-catenin (proteintech; 1:1000; USA) and phospho-γH2AX (Cell Signaling, 1:1000; USA), HMGB1 (abcam, 1:10000; UK) and p-chk1, p-chk2, p-cdc25C, ATRIP (Cell Signaling; 1:1000; USA), acetyl-H3 (Lys9) (absin, 1:5000; China), GFP (proteintech, 1:2000, USA). Finally, proteins were visualized with ECL and Kodak film without light.

### Immunofluorescent staining

Before or after IR exposure, cells or sections from xenografts were fixed with 4% paraformaldehyde for 20 min and permeabilized by 0.5% triton for 10 min. The cells were then soaked in normal goat serum for 30 min and incubated with primary antibodies: c-myc (proteintech; 1:50; USA), RAD51 (abcam; 1:1000; UK), KU80 (abcam; 1:1000; UK), β-catenin (proteintech; 1:50; USA), MDC1 (proteintech; 1:50; USA), and phospho-γH2AX (Cell Signaling (20E3); 1:250; USA) overnight at 4 °C. After washed by PBS for three times, the cells were then incubated with Alexa Flura® 488 conjugated, goat anti-rabbit IgG at room temperature for 2 h. Then the cells’ nuclei were stained with DAPI. Finally, images were obtained with a fluorescent microscope (Zeiss) and images were captured under the same exposure time.

### ChIP assays

Pierce Agarose ChIP Kit (Thermo; USA) was used to perform ChIP assays. Firstly, rKyse150 cells were cross-linked and lysed, and chromatin was sheared. Sheared chromatin-DNA mixture was then incubated with 4 μl IgG as negative control, 2 μl RNA polymerase II antibody as positive control, 5 μl beta-catenin (GTX; USA) and 5 μl TCF4 (Cell Signaling; USA) antibody overnight at 4 °C. PCR was amplified using eight primers shown in Supplementary Table S4.

### Cell transfection

For the reduction and induction of HMGB1 expression, GFP-tagged of human plenti/HMGB1, plenti/siHMGB1 and matched controls (plenti-Blank and Scrambled siRNA) plasmids were purchased from ABM (ABM, Canada). Cells were transfected with plasmids using DNAifectin Plus (ABM, Canada) according to the manufacturer’s protocol. Stably transfected cells were selected for 14 days in the presence of 2 μg/ml puromycin (ABM, Canada).

### Statistical analysis

Two-tailed Student’s *t*-test was used to determine the level of significance (*P < 0.05, **P < 0.01) by IBM SPASS statistics. All the quantitative data presented were the mean ± SD from at least three independent samples.

## Electronic supplementary material

Supplementary figure 1(TIF 3587 kb)

Supplementary figure 2(TIF 1557 kb)

Supplementary figure 3(TIF 4068 kb)

Supplementary figure 4(TIF 1442 kb)

Supplementary figure 5(TIF 2501 kb)

Supplementary figure 6(TIF 2278 kb)

Supplementary figure legends(DOCX 15 kb)

Supplementary table(DOCX 20 kb)
