# Supplementary figures and images for: Wnt signaling induces radioresistance through upregulating HMGB1 in esophageal squamous cell carcinoma
Source: Cell Death Dis. 2018 Mar 22;9(4):433. doi: 10.1038/s41419-018-0466-4 (PMC5864958; doi:10.1038/s41419-018-0466-4)

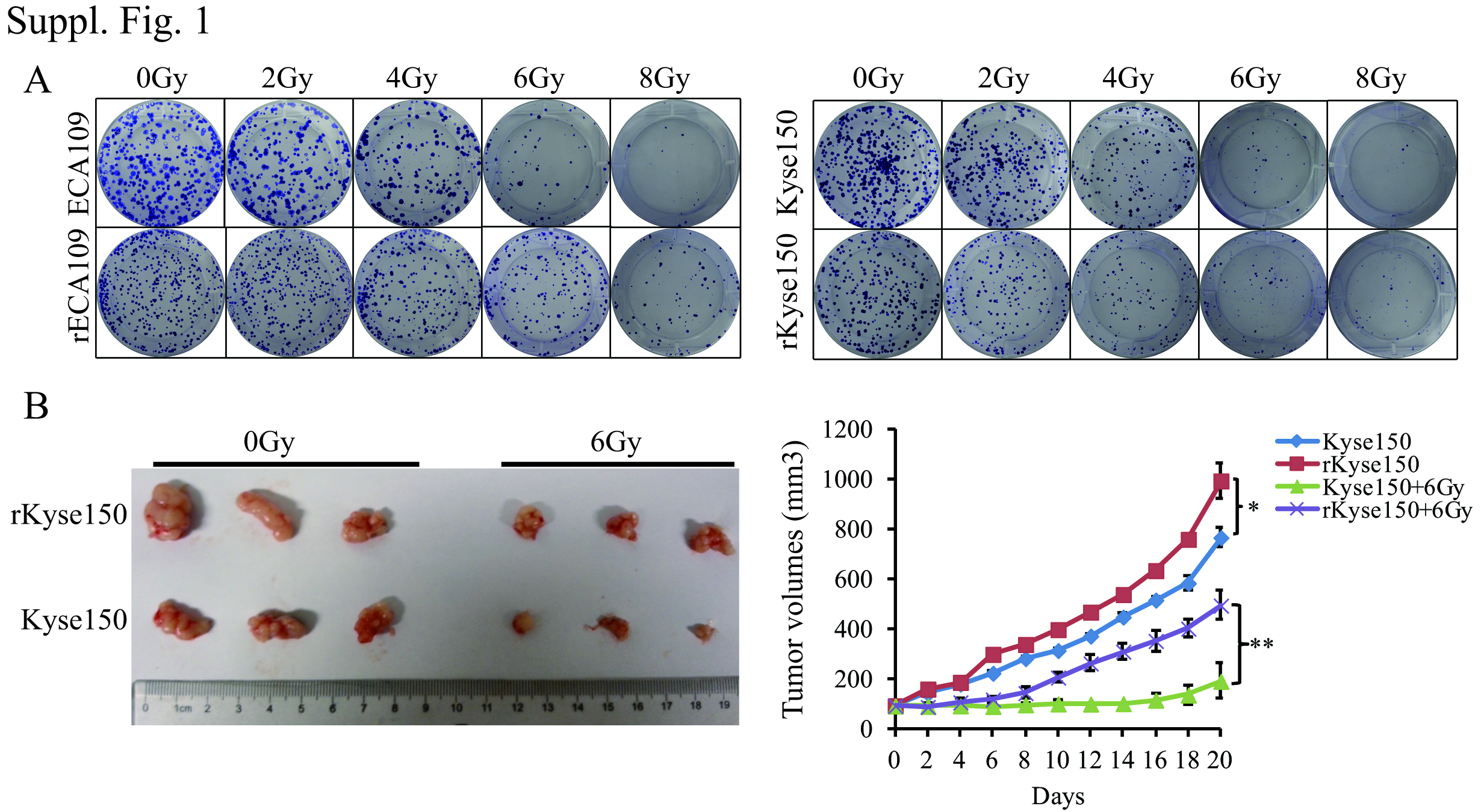

Supplement: Supplementary file 1 — Supplementary figure 1(TIF 3587 kb) [file 41419_2018_466_MOESM1_ESM.tif]

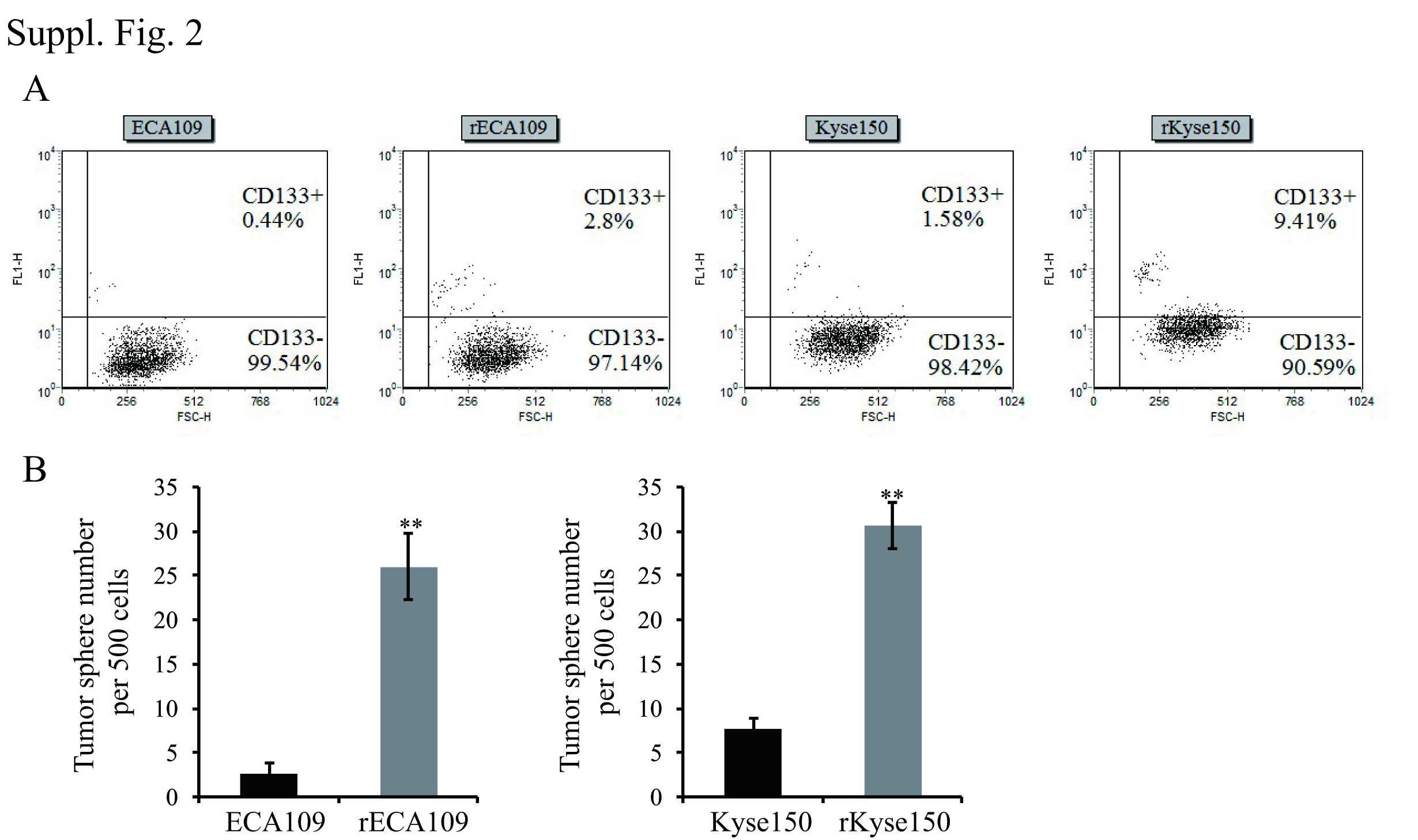

Supplement: Supplementary file 2 — Supplementary figure 2(TIF 1557 kb) [file 41419_2018_466_MOESM2_ESM.tif]

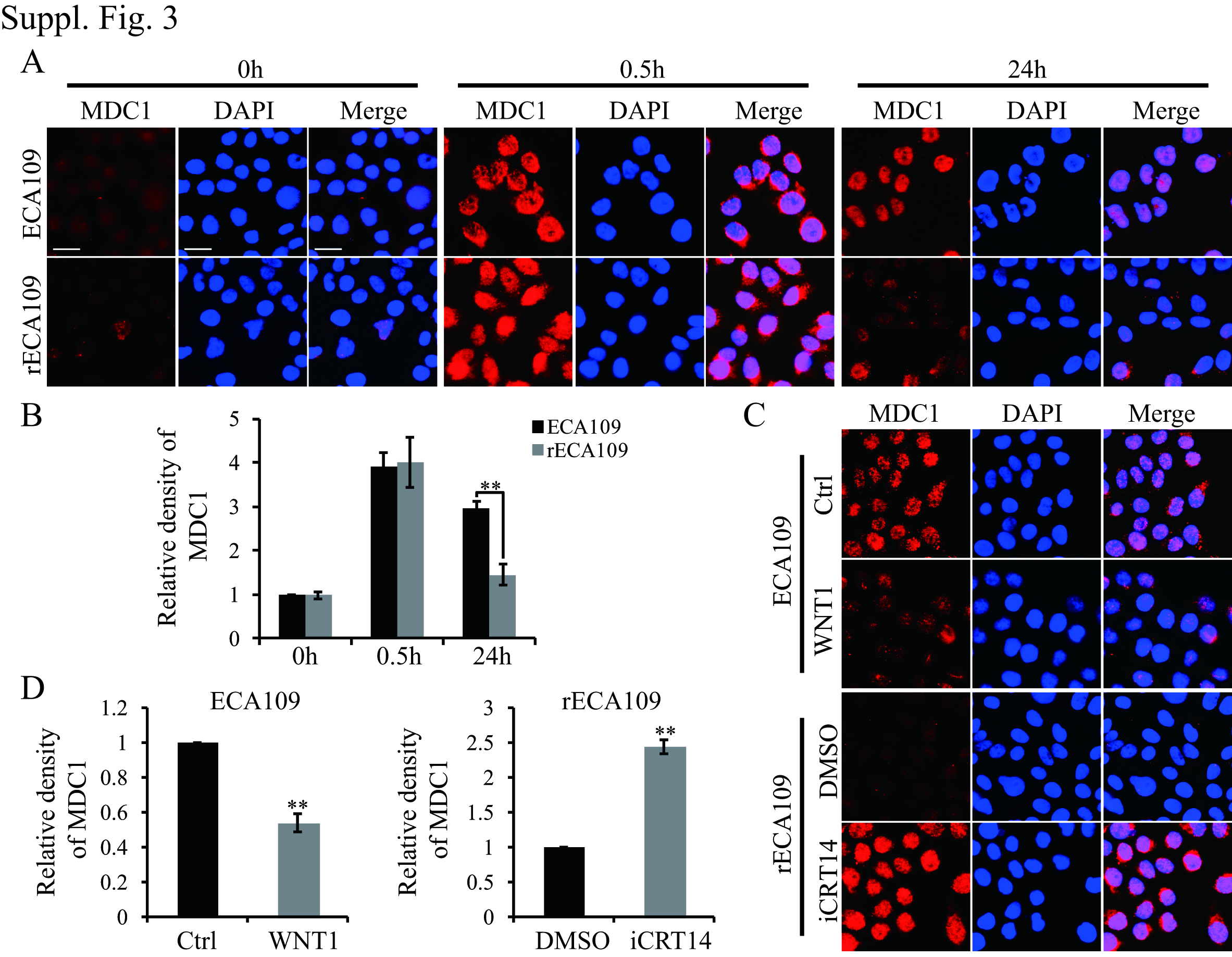

Supplement: Supplementary file 3 — Supplementary figure 3(TIF 4068 kb) [file 41419_2018_466_MOESM3_ESM.tif]

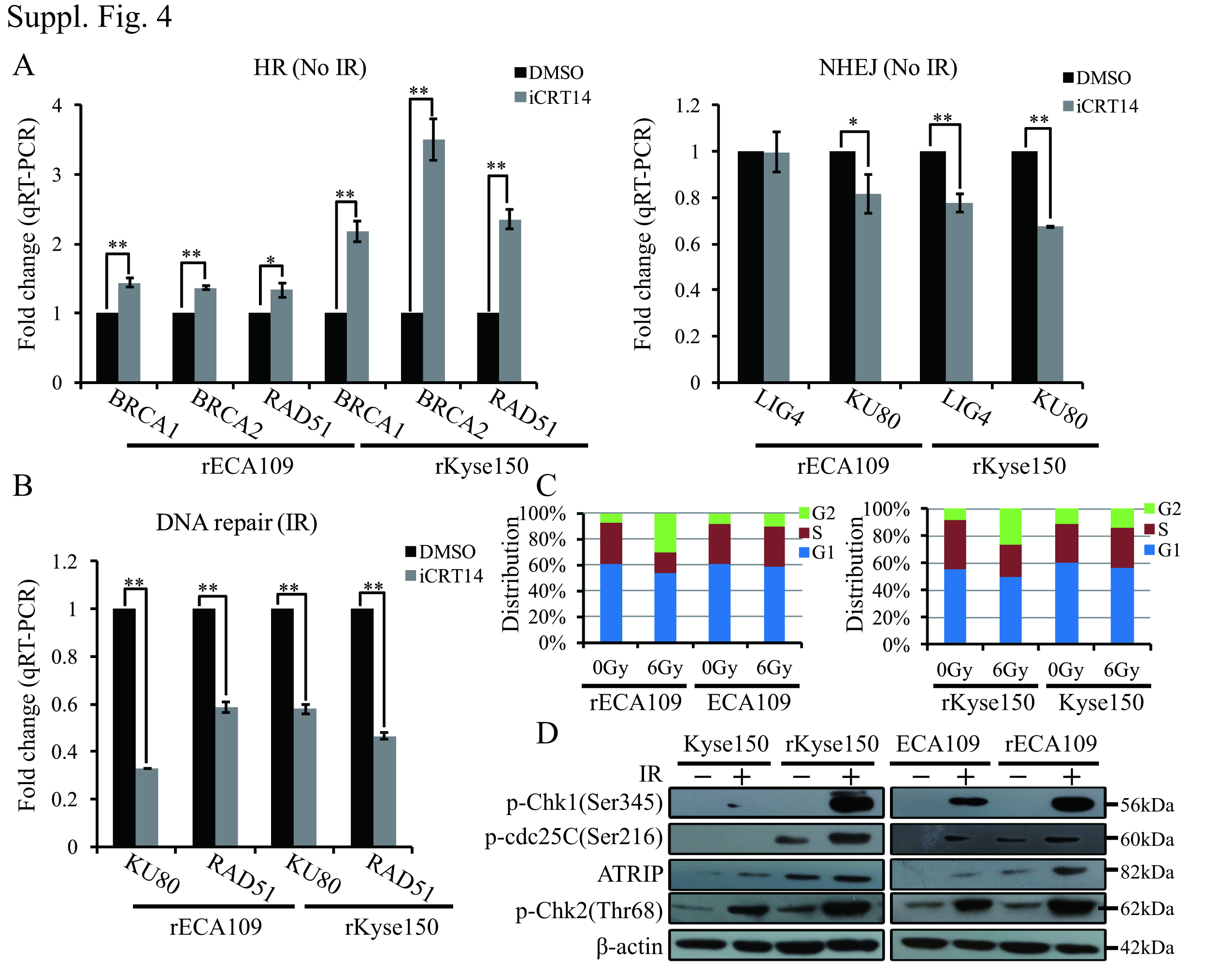

Supplement: Supplementary file 4 — Supplementary figure 4(TIF 1442 kb) [file 41419_2018_466_MOESM4_ESM.tif]

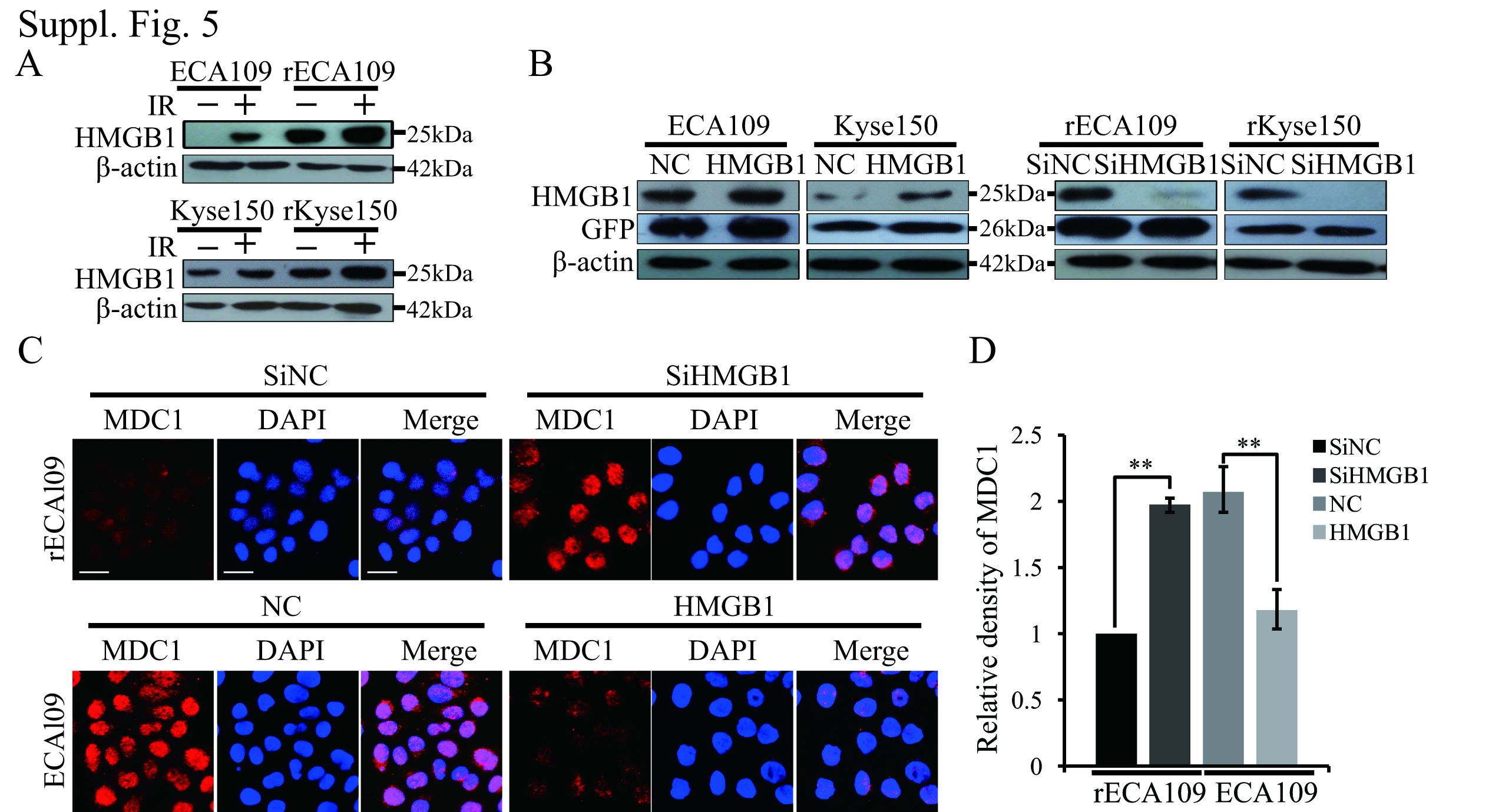

Supplement: Supplementary file 5 — Supplementary figure 5(TIF 2501 kb) [file 41419_2018_466_MOESM5_ESM.tif]

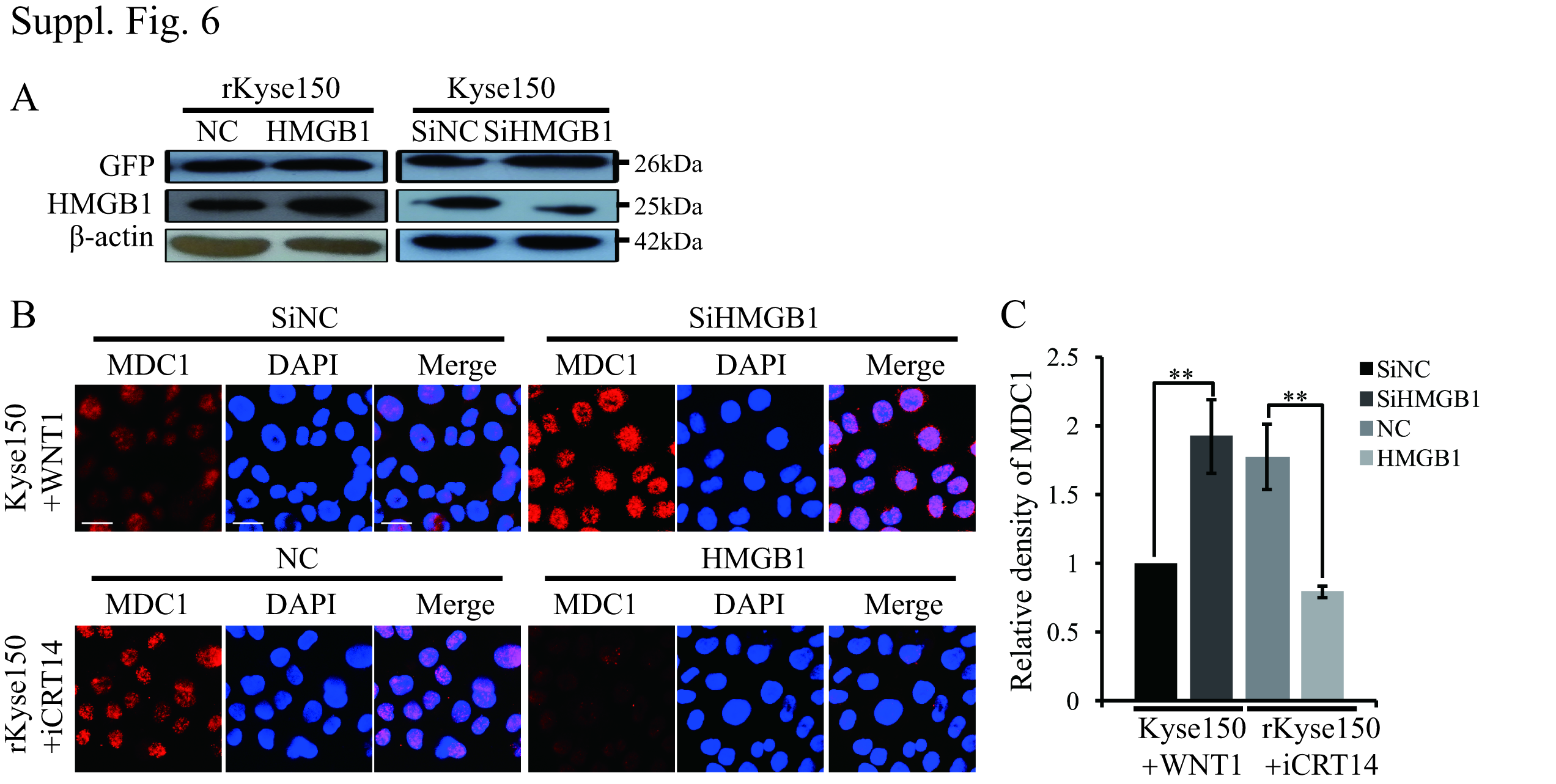

Supplement: Supplementary file 6 — Supplementary figure 6(TIF 2278 kb) [file 41419_2018_466_MOESM6_ESM.tif]
