# Supplementary material for: Wnt signaling induces radioresistance through upregulating HMGB1 in esophageal squamous cell carcinoma
Source: Cell Death Dis. 2018 Mar 22;9(4):433. doi: 10.1038/s41419-018-0466-4 (PMC5864958; doi:10.1038/s41419-018-0466-4)
Supplement: Supplementary file 7 — Supplementary figure legends(DOCX 15 kb) [file 41419_2018_466_MOESM7_ESM.docx]

**Supplementary Figure S1**

(A) Survival clonogenic assay. Parental and radioresistant cells were seeded into 6-well plates in a low density. Cells were then subjected to 0, 2, 4, 6 and 8Gy of IR. The colonies were then grown for 10 days. (B) Tumor survival curves (n=5). Tumor volume was recorded every other day and presented as mean ± SD. 21 days after IR, the tumors were removed. Except the mice dead after IR, 3 representative xenografts each group were captured and shown. *P < 0.05, **P < 0.01.

**Supplementary Figure S2**

(A) Flow cytometry of CD133 of ECA109, rECA109, Kyse150 and rKyse150. CD133+ fractions were assessed by FACS. (B) The sphere numbers of ECA109, rECA109, Kyse150 and rKyse150 per 500 cells. Mean±SD, N=3, *P<0.05, **P<0.01.

**Supplementary Figure S3**

(A) IF staining of MDC1 of radioresistant cells and parental cells. Radioresistant and parental cells were treated with IR (6Gy). Before IR exposure or 0.5h, 24h after IR exposure (6Gy), tumor were fixed for IF staining for MDC1. Scale bars=40μm. (B) Quantification of MDC1 by image pro plus software. Results were from Figure A. Mean±SD, N=3, *P<0.05, **P<0.01. (C) IF staining of MDC1 of cells with or without WNT1/iCRT14 pretreatment. Parental cells pretreated by WNT1 (100 ng/ml) (cells without WNT1 pretreatment as control) and radioresistant cells pretreated with iCRT14 (25μM) (cells with DMSO pretreatment as control) were subjected to IR (6Gy). 24h after IR (6Gy), IF staining were performed. Scale bars=40μm. (D) Quantification of MDC1by image pro plus software. Results were from Figure C. Mean±SD, N=3, *P<0.05, **P<0.01.

**Supplementary Figure S4**

(A) qRT-PCR analysis of NHEJ-related and HR-related genes. rECA109 and rKyse150 cells were treated with iCRT14 (25μM for 24h) and analyzed for expression of NHEJ and HR repair genes in the absence of using qRT-PCR. Mean±SD, N=3, *P<0.05, **P<0.01. (B) qRT-PCR analysis of KU80 and RAD51. Both KU80 and RAD51 were down-regulated by iCRT14 treatment after IR treatment. Mean±SD, N=3, *P<0.05, **P<0.01. (C) Cell cycle analysis of radioresistant cells and parental cells both before IR and 0.5h after IR. (D) Western blotting analysis of representative proteins of DNA damage checkpoint response. Parental and radioresisant cells were collected to perform Western blotting both before IR and 0.5h after IR.

**Supplementary Figure S5**

(A) Western blotting analysis of HMGB1. Western blotting was performed before IR treatment and 6h after IR (6Gy). (B) Western blotting analysis of HMGB1. The change levels of HMGB1 of the parental cells transfected with plenti/HMGB1 and the radioresistant cells transfected with plenti/SiHMGB1 were assessed by western blotting. (C) IF staining of MDC1. Stable-transfected ECA109 cells (plenti/HMGB1 and plenti/NC) and rECA109 cells (plenti/SiHMGB1 and plenti/SiNC) were collected to analyze the MDC1 levels 24h after IR using IF staining. Scale bars=40μm. (D) Quantification of MDC1 by image pro plus software. Results were from Figure C. Mean±SD, N=3, *P<0.05, **P<0.01.

**Supplementary Figure S6**

(A) Western blotting analysis of HMGB1. The change levels of HMGB1 of Kyse150 transfected with plenti/SiHMGB1 and rKyse150 transfected with plenti/HMGB1 were assessed by western blotting. (B) IF staining of MDC1. 24h after WNT1 treatment for Kyse150/SiHMGB1 and Kyse150/SiNC cells and iCRT14 treatment for rKyse150/HMGB1 and rKyse150/NC cells, cells were exposed to IR. 24h after IR, IF staining of MDC1 were performed to assess the DNA damage levels. Scale bars=40μm. (C) Quantification of MDC1 by image pro plus software. Results were from Figure B. Mean±SD, N=3, *P<0.05, **P<0.01.
