# Supplementary material for: Wnt signaling induces radioresistance through upregulating HMGB1 in esophageal squamous cell carcinoma
Source: Cell Death Dis. 2018 Mar 22;9(4):433. doi: 10.1038/s41419-018-0466-4 (PMC5864958; doi:10.1038/s41419-018-0466-4)
Supplement: Supplementary file 8 — Supplementary table(DOCX 20 kb) [file 41419_2018_466_MOESM8_ESM.docx]

Supplementary Table S1 Tumor growth delay after IR treatment

|  | ECA109 | rECA109 | P value |
| --- | --- | --- | --- |
| Tumor growth delay | 81% ± 7% | 50% ± 4% | 0.007 |

|  | Kyse150 | rKyse150 | P value |
| --- | --- | --- | --- |
| Tumor growth delay | 83% ± 6% | 47% ± 7% | 0.005 |

Supplementary Table S2 Parameters of clonogenic survival assay fitting to a multi-target model

|  | ECA109/NC | ECA109/HMGB1 | P value |
| --- | --- | --- | --- |
| D0 | 1.93 ± 0.11 | 2.69 ± 0.13 | 0.004 |
| Dq | 2.57 ± 0.1 | 3.58 ± 0.29 | 0.010 |

|  | rECA109/SiNC | rECA109/SiHMGB1 | P value |
| --- | --- | --- | --- |
| D0 | 2.81 ± 0.11 | 2.19 ± 0.11 | 0.004 |
| Dq | 3.90 ± 0.23 | 2.59 ± 0.12 | 0.002 |

|  | Kyse150/NC | Kyse150/HMGB1 | P value |
| --- | --- | --- | --- |
| D0 | 2.12 ± 0.06 | 2.99 ± 0.04 | 0.000 |
| Dq | 2.60 ± 0.06 | 3.09 ± 0.16 | 0.017 |

|  | rKyse150/NC | rKyse150/SiHMGB1 | P value |
| --- | --- | --- | --- |
| D0 | 2.75 ± 0.14 | 1.98 ± 0.03 | 0.001 |
| Dq | 3.58 ± 0.09 | 2.66 ± 0.05 | 0.000 |

Supplementary Table S3 Primer sequences of target genes

| Gene | Forward primer(5’-3’) | Reverse primer(5’-3’) |
| --- | --- | --- |
| β-actin | AGCGAGCATCCCCCAAAGTT | GGGCACGAAGGCTCATCATT |
| HMGB1 | TATGGCAAAAGCGGACAAGG | CTTCGCAACATCACCAATGGA |
| BRCA1 | GAAACCGTGCCAAAAGACTTC | CCAAGGTTAGAGAGTTGGACAC |
| BRCA2 | ACAAGCAACCCAAGTGTCAAT | TGAAGCTACCTCCAAAACTGTG |
| RAD51 | GGTCTGGTGGTCTGTGTTGA | GGTGAAGGAAAGGCCATGTA |
| KU80 | GTGCGGTCGGGGAATAAGG | GGGGATTCTATACCAGGAATGGA |
| LIG4 | AGCAAAAGTGGCTTATACGGATG | TGAGTCCTACAGAAGGATCATGC |
| CHD3 | CCGTCAGCATTGGGTGTGAA | TCTTGCGTTTTCGGGGTTTTC |
| CHD4 | TGAGGGCAGCGACTATACTCC | GAGCAGATGATTTAGGCTCCTTT |
| MTA1 | ACGCAACCCTGTCAGTCTG | GGGCAGGTCCACCATTTCC |
| Tip49 | AGGTGAAGAGCACTACGAAGA | CTACTATGACGCCACATGCCT |
| SiRT6 | CCCACGGAGTCTGGACCAT | CTCTGCCAGTTTGTCCCTG |
| Fe65 | GGAGGGGACGTTGACCTTC | TTTTGTGGTAAGAGAGCTGACG |
| ACF1 | CTGCTACACCGAAAGCCGTT | GCACAGAATGGTTCGTTCAAAAA |

Supplementary Table S4 primers of ChIP assays

| Primers | Forward primer | Reverse primer |
| --- | --- | --- |
| 1 | AATCTTTACAACCCTGTGGTCCCCTC | TCGATGATATTAAAATTTGACGATTAGTTGAATATGGCACTATCAATA |
| 2 | ATGGCCGTGCCAGTACAAAG | CGGACTCAGGGATTATAGTCTTGG |
| 3 | TGATTAGTAGAGGGAAGCAGAGGAT | TTTAGCCTGAGCAGACCACG |
| 4 | ACATTTGGAGGAATTGGTTGTTGGA | TGCATCCATGTAACTGCTCTAGGA |
| 5 | CAGCCTGTGGATTTGGGCAC | ACAAAGCACTGTCTTAACTTCCTGG |
| 6 | GGCCTCACAAACATTTCCTGCT | CGAGCCCCTCAAAGGTCTCT |
| 7 | TTTAAACCCGGCTGGCTGGA | GGAGCGAAAAGGGCCTGACT |
| 8 | GCGTGAATGTGGGGCAAGAA | TCATTGGCCCGATACCTCCC |
